# Sero-Prevalence of SARS-CoV-2 Antibodies in High-Risk Populations in Vietnam

**DOI:** 10.3390/ijerph18126353

**Published:** 2021-06-11

**Authors:** Tasnim Hasan, Thach Ngoc Pham, Thu Anh Nguyen, Hien Thi Thu Le, Duyet Van Le, Thuy Thi Dang, Trang Dinh Van, Yen Ngoc Pham, Ha Viet Nguyen, Giang Linh Tran, Van Thi Cam Nguyen, Thanh Trung Nguyen, Viet Quang Truong, Than Huu Dao, Chung Thanh Le, Nam Tan Truong, Hoang Trung Vo, Phuc Thanh Le, Thao Thanh Nguyen, Vinh Van Luu, Vinh Dai Nguyen, Brett G. Toelle, Guy B. Marks, Greg J. Fox

**Affiliations:** 1 Faculty of Medicine and Health, The University of Sydney, Sydney 2006, Australia; tasnim.hasan@sydney.edu.au (T.H.); brett.toelle@sydney.edu.au (B.G.T.); 2 The Woolcock Institute of Medical Research, Glebe 2037, Australia; thuanh.nguyen@sydney.edu.au (T.A.N.); hien.le@sydney.edu.au (H.T.T.L.); yen.phamngoc@sydney.edu.au (Y.N.P.); vietha.nguyen@sydney.edu.au (H.V.N.); tranlinhgiang96@gmail.com (G.L.T.); van.nguyencam@sydney.edu.au (V.T.C.N.); thanh.nguyentrung@sydney.edu.au (T.T.N.); g.marks@unsw.edu.au (G.B.M.); 3 National Hospital for Tropical Diseases, Hanoi 12319, Vietnam; phamngocthachnhtd@gmail.com (T.N.P.); duyetibt@gmail.com (D.V.L.); dangthuy.nhtd@gmail.com (T.T.D.); vandinhtrang.nhtd@gmail.com (T.D.V.); 4 Hanoi Center for Disease Control (CDC), Hanoi 11512, Vietnam; bstruongquangviet@gmail.com (V.Q.T.); daohuuthan@gmail.com (T.H.D.); 5 Da Nang Centre for Disease Control, Da Nang City 50213, Vietnam; lethanhchung65@yahoo.com (C.T.L.); namtrt.aidsdn@gmail.com (N.T.T.); 6 Quang Nam Centre for Disease Control, Quang Nam 51108, Vietnam; vthoangsr@gmail.com; 7 Da Nang Lung Hospital, Da Nang City 50606, Vietnam; mslephuctb@yahoo.com.vn; 8 Quang Nam Pham Ngoc Thach Hospital, Quang Nam 51110, Vietnam; thaomaidao@yahoo.com.vn (T.T.N.); luuvanvinh@gmail.com (V.V.L.); 9 Hoa Vang District Health Center, Da Nang City 50807, Vietnam; daivinh75@gmail.com; 10 Sydney Local Health District, Sydney 2050, Australia; 11 South Western Sydney Clinical School, The University of New South Wales, Liverpool 2170, Australia

**Keywords:** COVID-19, SARS-CoV-2, seroprevalence, Vietnam

## Abstract

As a response to the coronavirus disease 2019 (COVID-19) pandemic, Vietnam enforced strict quarantine, contact tracing and physical distancing policies resulting in one of the lowest numbers of individuals infected with severe acute respiratory syndrome-coronavirus-2 (SARS-CoV-2) globally. This study aimed to determine the prevalence of SARS-CoV-2 antibody positivity among high-risk populations in Vietnam. A prevalence survey was undertaken within four communities in Vietnam, where at least two COVID-19 cases had been confirmed. Participants were classified according to the location of exposure: household contacts, close contacts, community members, and healthcare workers (HCWs) responsible for treating COVID-19 cases. Participants completed a baseline questionnaire and SARS-CoV-2 IgG antibodies were quantified using a commercial assay. A total of 3049 community members and 149 health care workers consented to the study. Among 13 individuals who were seropositive (0.4%), five household contacts (5/27, 18.5%), one close contact (1/53, 1.9%), and seven community members (7/2954, 0.2%) had detectable SARS-CoV-2 antibodies. All HCWs were negative for SARS-CoV-2 antibodies. Participants were tested a median of 15.1 (interquartile range from 14.9 to 15.2) weeks after exposure. Our study found a low prevalence of SARS-CoV-2 antibodies in high-risk communities and healthcare workers in communities in Vietnam with known COVID-19 cases.

## 1. Introduction

Vietnam is a populous Southeast Asian country, bordering China. By December 2020, the country had reported among the lowest number of cases of infection with severe acute respiratory syndrome-coronavirus-2 (SARS-CoV-2) globally [1]. Prompt border closures, quarantine of returning travellers, and strict isolation of proven cases as well as their first- and second-generation contacts contributed to the rapid containment of the virus [2]. As of 1 December 2020, 1351 laboratory confirmed cases of coronavirus disease 2019 (COVID-19) had been reported, 51% of whom were returned travellers in quarantine [3]. By this date 35 deaths were recorded as being due to COVID-19 [1]. However, it is possible that the number of reported cases may be an underestimate of the true incidence of disease. This is because some people with the infection may not have been tested as they did not have symptoms, did not seek care, or were not able to access a virus-detection test. Serological tests measure the antibody response to the virus, with a response evident from 10–14 days after the onset of infection [4,5]. Sero-surveys in potentially exposed populations can be used to evaluate the true cumulative incidence of infection with SARS-CoV-2 and, by comparison with the reported incidence rate, to estimate the case detection rate [6].

The seroprevalence of SARS-CoV-2 antibodies varies substantially between settings [5,7], reflecting the variation in countries’ experience of the pandemic. Countries implementing successful public health measures to reduce transmission—including physical distancing, effective quarantining of high-risk individuals and strict border controls—have reported a low prevalence of SARS-CoV-2 antibodies (seroprevalence) in the population. For example, seroprevalence rates of less than 1% in the general population were reported in Greece, Malaysia, and in Sydney in mid-2020 [8,9,10]. In contrast, in high transmission settings, including Northern Europe and North America, the seroprevalence of infection in sampled populations had been reported to be as high as 12.5% [11,12,13]. In Switzerland, due to low levels of confirmatory testing, for every confirmed SARS-CoV-2 case in the community, antibody testing revealed that a further 11.6 cases of SARS-CoV-2 infection had been undiagnosed [12]. Health care workers (HCWs) exposed to patients with COVID-19 are often reported to be at the highest risk of infection, with antibody seroprevalence reported between 6.4% and 24.4% [14,15,16].

Vietnam shares a normally porous, 1300 km northern border with China. The first case of COVID-19 was diagnosed in Vietnam on 23 January 2020, in a returned traveller from Wuhan, China. Within eight weeks, Vietnam had closed its national borders, introduced quarantine procedures, closed all schools and businesses, implemented physical distancing policies, and utilised comprehensive public health messaging to the population [17]. All suspected and confirmed cases were required to enter mandatory quarantine at public facilities following a risk assessment.

The presence of undetected transmission in Vietnam is unknown. This study aimed to measure the prevalence of a serological response to SARS-CoV-2 in communities where cases of COVID-19 were reported and among household contacts and healthcare workers exposed to patients known to have COVID-19.

## 2. Materials and Methods

### 2.1. Study Design and Setting

A cross-sectional study was performed in three provinces of Vietnam in which clusters of SARS-CoV-2 cases had been detected. Vietnam is a middle-income country in Southeast Asia with a population of 96 million. Within each of its 63 provinces, healthcare is delivered by the provincial government’s Department of Health with support from the national Ministry of Health. Each province is further subdivided into districts, communes, and sub-communes. Sub-communes usually have a population of between 500 and 2000 people.

From January to July, approximately 70% of confirmed cases had acquired the virus overseas [17]. Following 99 days with no detected community transmission of COVID-19, on 25 July 2020, Vietnam reported a new case of COVID-19 in a patient presenting with an acute respiratory illness to the Da Nang Provincial Hospital. This outbreak in central Vietnam quickly spread into the local community and 13 other provinces. Despite an increase in community transmission, the outbreak was effectively controlled within a 40-day period, with a coordinated public health response. Figures S1–S3 shows the number of cases detected in affected provinces over time.

### 2.2. Site Selection

Sub-communes were eligible for inclusion in this study if they had at least two cases of COVID-19 confirmed, based upon real-time reverse transcriptase polymerase chain reaction (rRT-PCR) testing of nasal/throat swabs. Sites were selected to reflect both urban and rural settings and to include sub-communes with the highest cases numbers (Tables S1 and S2). Finally, four sub-communes were selected. These were in northern Vietnam: (i) Hoi sub-commune in Ha Loi commune, Me Linh district, Hanoi Capital (where five cases of known COVID-19 were diagnosed in April 2020); and in central Vietnam: (i) Giao Ai sub-commune in Dien Hong commune, Dien Ban, Quang Nam Province (three cases of known COVID-19 in August 2020); (ii) Luu Minh sub-commune in Ha Lam Town, Quang Nam (seven cases of known COVID-19 between July and August 2020); (iii) Le Son Nam sub-commune in Hoa Tien commune, Da Nang (eight cases in August 2020).

### 2.3. Study Population

Inclusion criteria: All people residing in each sub-commune during the period of the local COVID-19 outbreak who were aged 5 years and older and who were capable of giving consent, or having consent given by a guardian (for children < 16 years), were eligible to participate.

Exclusion criteria: Individuals were excluded if they were unable or refused to provide consent, were less than five years of age, or if they were a household/community member who had not stayed in the same house/sub-commune during the infectious period. Individuals who were documented as PCR-positive for COVID-19 during the outbreaks were excluded from testing.

The names and addresses of household contacts and close contacts of COVID-19 cases were obtained from the local Centres for Disease Control (CDC). Enrolled participants were classified as close contacts, household contacts, or general community members. Close contacts of COVID-19 cases were those who been within a two-meter distance of the COVID-19 case for at least 15 min, or had been present in the same room for at least two hours during the infectious period (that is, from 48 h prior to symptom onset or diagnosis until the person with confirmed COVID-19 was placed in isolation). Household contacts of a COVID-19 case were those who were not close contacts, but who were living in the same dwelling and sharing meals or the kitchen with a COVID-19 case during the infectious period. General community members were those who were not close or household contacts of the COVID-19 case, but were living in the same sub-commune as the COVID-19 case. The sample size required to measure the prevalence of seropositivity within each commune, with a confidence interval width of ±5%, assuming a household size of three and household sero-positivity of up to 50%, was 311 households or 933 participants.

### 2.4. Health Care Workers

HCWs were enrolled at two health care facilities: Da Nang Lung Hospital, a provincial hospital in Da Nang, and Hoa Vang District Hospital, a district hospital in Da Nang. All HCWs enrolled were directly involved in the care of COVID-19 cases. Doctors, nurses, allied health workers, laboratory staff, and any other ancillary staff who were involved in patient care were included.

### 2.5. Study Questionnaire

Interviews were conducted with each study participant between September and November 2020. Data were collected about participants’ age, gender, contact status and history of infective symptoms, travel outside, or inside Vietnam since 1 January 2020, occupation, comorbidities, and medications. Frequency of travelling between Da Nang and Quang Nam, two neighboring provinces in central Vietnam in which the second outbreak occurred, was also recorded as a risk factor. The proximity, duration, and number of exposures with COVID-19 cases within the infectious period were also recorded. Surveys were conducted and recorded using a REDCap database.

### 2.6. Serology Testing

Blood was drawn for serological testing a median of 15.1 weeks (IQR 14.9–15.2) after confirmation of a COVID-19 case in the community or household. Where the result of the assay was indeterminate, the test was repeated. We used the Elecsys Anti-SARS-CoV-2 serology assay on the cobas platform (Roche Diagnostics International Ltd., Rotkreutz, Switzerland). This assay has been shown to have a sensitivity and specificity of 96.8% and 99.8%, respectively, for samples taken at least 14 days after symptom onset [18]. The test was performed in accordance with the manufacturer’s instructions.

### 2.7. PCR Testing for SARS-CoV-2

Participants, including HCWs who were symptomatic at the time of the survey, were offered PCR for SARS-CoV-2 if they met local testing criteria [19].

### 2.8. Analysis

Descriptive statistics were calculated, stratified by cluster, for each category of individual—household contact, close contact, community member, and HCW.

### 2.9. Ethical Issues

Ethical approval was obtained from the Human Research Ethics Committees of the University of Sydney (HREC 2020/415) and Biomedical Research Ethics Committee of the National Hospital for Tropical Diseases (No. 10/HDDD-NDTU and No. 18/HDDD-NDTU). Consent was documented electronically using a tablet computer. In accordance with local expectations, all COVID-19 patients and other participants were provided with monetary compensation for their participation, equivalent to approximately USD 4.30 and USD 2.20, respectively.

## 3. Results

A total of 3049 individuals of 3747 (81.4%) community members who met inclusion criteria for the study, provided consent for the study, and blood samples for serology were collected from 3034 of these (99.5% of those consenting and 81.0% of those eligible). The demographic, epidemiological, and clinical features of study participants, classified by sub-commune, are presented in Table 1. Most participants (2969) were classified as community members (97.4%), while 27 were household contacts (0.9%), and 53 were close contacts (1.7%). The median age of the population was 37 (interquartile range (IQR) 19–53). There were 429 (14.1%) individuals who reported a history of smoking and 362 (11.9%) had a history of other comorbidities.

Only one study participant had travelled outside of Vietnam since January 2020. Almost 20% had travelled within Vietnam since the onset of the pandemic. Most local travel had been conducted using private vehicles.

### 3.1. Symptoms and Actions

Only 162 (5.3%) study participants had reported any relevant symptoms during the duration of the local COVID-19 outbreak. The median number of symptoms reported was one (IQR 1–2). The most common symptoms were sore throat, rhinorrhea, headache, and fatigue. When symptoms became evident, the most common action was to seek advice from the local pharmacist, although many had waited for their symptoms to resolve spontaneously. Most individuals reported increased hand washing, the use of face masks, and a reduction in attending social gatherings, over the course of the year (Table 1).

### 3.2. Details of Exposure

Most exposure to COVID-19 cases took place at home. Other settings in which community members had exposure to COVID-19 patients included cafes, the marketplace, and at work (Table 2). A small number of participants were exposed to COVID-19 patients at health care facilities, including one individual who had exposure at a dialysis centre over several days. The number of COVID-19 contacts varied by sub-commune, with those who were household contacts in the Luu Minh commune having an average of 4.3 household COVID-19 contacts. For those who reported a history of close contact, most had contact with just one or two COVID-19 cases. The duration of exposure with confirmed COVID-19 cases varied from 15 min to 24 h, with longer exposure generally reported by those who were household contacts.

### 3.3. Health Care Workers

One hundred and forty-eight health care workers who had direct exposure to COVID-19 cases between two hospitals were included (Table 3). The median number of hours of contact with a confirmed case per day was 6 h, and healthcare workers reported a median of 40 days (IQR 30–48.5) working in a healthcare setting where patients with COVID-19 were present. Over half of the HCWs were nurses and a quarter were doctors. Twenty-nine (19.5%) individuals reported any relevant symptoms during the local COVID-19 outbreak. The median number of symptoms was two (IQR 1–2). Only 13 (8.7%) healthcare workers reported comorbidities.

### 3.4. Serology

Only 13 of 3034 participants (0.4%) had detectable IgG antibodies to SARS-CoV-2. The sero-positive participants included five of 27 (18.5%) household contacts, one of 53 (1.9%) close contacts, and seven of 2954 (0.2%) community members (Table 4). Of 148 HCWs who had serology performed, none had detectable antibodies (Table 3).

The median age of sero-positive participants was 35 (IQR 22–49), and seven (53.8%) were female (Table 5). Three sero-positive participants were under 15 years of age. Threeother sero-positive individuals were from the same household. The remaining ten sero-positive participants were each from different households. No seropositive individuals had travelled outside of Vietnam, and none of those in north Vietnam had travelled to Da Nang or Quang Nam provinces. Two people with a positive test had travelled to other areas in Vietnam, in three trips—all of which were by bus, however none had travelled to areas where there were known COVID-19 outbreaks.

Six of the 13 people with a positive serological test, including the five close contacts and one household contact, were aware of exposure to a known COVID-19 case. One individual had contact with four COVID-19 cases during the infectious period. Another sero-positive participant reported contact with two infectious COVID-19 cases and four sero-positive individuals had contact with one COVID-19 case while the contact was infectious. The average duration of contact with known COVID-19 cases while infectious was 6.45 h. One sero-positive individual had symptoms at the time of community exposure (headache and conjunctivitis) during the community outbreak and exposure in the Hoi District in northern Vietnam in April. These six household contacts and close contacts were all quarantined after exposure. However, the remaining seven sero-positive participants, who were classified as community contacts, were not aware of their exposure to a known COVID-19 case and local policy did not require compulsory facility-based quarantine for community contacts. However, people living in the same sub-commune as a COVID-19 case (that is, community contacts) were encouraged to remain in home isolation.

Sixty-seven individuals had PCR performed as part of the study, due to the presence symptoms at the time of screening. All PCR tests were negative.

## 4. Discussion

This survey of populations at high risk of infection with COVID-19 in Vietnam revealed a low prevalence of SARS-CoV-2 antibodies, establishing that the local public health measures have been effective in curtailing the spread of COVID-19 within the community. Although the prevalence of antibodies to SARS-CoV-2 was high in household contacts of confirmed COVID-19 cases, the overall prevalence of antibodies in the general population in these four high-risk communities was just 0.4%. Remarkably, all tested HCWs who worked in facilities managing COVID-19 patients had undetectable antibodies to SARS-CoV-2.

The sero-prevalence of SARS-CoV-2 antibodies among people who were residents of the sub-communes in which confirmed COVID-19 cases had been diagnosed, but were not household or close contacts, was found to be just 0.2%. Most published serological surveys have reported the prevalence of SARS-CoV-2 antibodies in HCWs, household, and close contacts [7,20,21]. Only a few studies have evaluated community transmission and in high-prevalence European contexts the community prevalence has been reported to be 1.6–3.9% [13,22], compared with 8.3–31.4% in household and other contacts [13,22]. The prevalence of antibodies in the community in Vietnam is substantially lower than that in other settings, suggesting that policies of isolating individuals with COVID-19 at the time of diagnosis, and policies to encourage community members to remain in isolation at home, have been effective in preventing the spread of infection into the community.

Transmission within households is an important factor in the spread of COVID-19. In this study 18.5% of household contacts were sero-positive for SARS-CoV-2 antibodies. Other studies have found the household prevalence of SARS-CoV-2 infection to be between 5.9 and 31.4% [7,13]. However, it is noteworthy, that in two of the sub-communes we investigated there were no household contacts with detectable antibody levels. In Vietnam, household contacts also underwent compulsory facility-based quarantine and this may have been an important factor in controlling ongoing transmission and community spread of infection.

In the current study, only one close contact had detectable SARS-CoV-2 antibodies. Few studies differentiate close contacts from household contacts. Other studies have found the prevalence of SARS-CoV-2 antibodies between 1.3 and 13.5% [7,13] in close contacts who were not household contacts. The lower transmission in close contacts compared to household contacts suggests that the duration of exposure is an important factor in the transmission of SARS-CoV-2.

The overall prevalence of SARS-CoV-2 antibodies in Vietnam (0.4%), is similar to other countries, such as Greece, Malaysia, and Australia, which had strict border control and local policies early in 2020 [8,9,10]. However, these studies were published early in 2020, before experiencing progressive outbreaks later in the year, whereas the current study has found a low prevalence of antibodies by late 2020 in Vietnam. The overall antibody prevalence was highest in Hoi, one of the earliest communities affected by COVID-19 in Vietnam, and lower seroprevalence in other communities. This may reflect improvements in public health responses in Vietnam as the pandemic evolved.

In this study, there were no HCWs with detectable antibodies to SARS-CoV-2, despite all having confirmed occupational exposure with COVID-19 cases. All HCWs reported wearing personal protective equipment (PPE) during exposure. There are only a limited number of settings where HCWs working directly with COVID-19 patients have remained sero-negative [23,24]. In Vietnam HCWs were assigned to COVID wards exclusively for a three to four-week period, and provided with accommodation and adequate PPE. After completing work in COVID-19 wards, HCWs completed a mandatory two-week quarantine period before being released back into the community. In health care settings in other countries, the prevalence of SARS-CoV-2 antibodies has been as high as 8.7–17.1% [14,25,26], with one of the biggest risks for HCW infection being household transmission [14,25,26]. However, studies have suggested that HCWs who have adequate protective equipment are less likely to acquire infection or be sero-positive [27,28,29]. The current findings may suggest that when the burden of infection is controlled nationally, this also becomes an effective strategy to protect frontline workers, by limiting household transmission and through the adequacy of PPE.

The findings of this study confirm that Vietnam’s public health strategy has been effective in suppressing two outbreaks of COVID-19, despite both having different transmission dynamics. During the first outbreak 70% of cases were imported from overseas [17], whereas during the second outbreak in Vietnam, the extent of community transmission was approximately 59% [3]. The self-reported increase in hand washing and mask wearing also affirm that educational strategies were effective in involving the Vietnamese community as part of the public health response.

Strengths of this study include the high participation rate, although this was achieved through monetary incentives, this allows a much more complete understanding of transmission in an entire community affected by COVID-19.

Limitations of this study include the delay in testing antibody status after exposure. Some guidelines suggest antibody testing should be performed within six weeks after exposure [5] and that antibodies can diminish with time, in particular in asymptomatic cases [30]. Despite this, the highest prevalence of sero-positivity was in the community where the outbreak was more than six months ago. Another limitation is that reporting of symptoms, adherence to public health actions and exposure to cases, may have been affected by recall-bias. Finally, the study does not assess adequacy and availability of PPE in protecting HCWs. Further study, looking at the adequacy and use of PPE will be important to help frame future infection control policy. Whilst case numbers remained low throughout 2020, by May 2021, evidence has emerged of community transmission with a potential SARS-CoV-2 variant of concern in Vietnam. Progress of this outbreak will determine if Vietnam can continue to hold the position as one of the few countries in the world which has controlled the COVID-19 pandemic.

## 5. Conclusions

Although not immune to further outbreaks and the economic and social consequences of the COVID-19 pandemic, the strict lockdown in Vietnam has prevented widespread community transmission of SARS-CoV-2. This study confirms that by late 2020, the prevalence of SARS-CoV-2 antibodies in the community remained low.

## Figures and Tables

**Table 1:** Demographic profile and characteristics of all sub-communes.

Characteristics	Total	Hoi Commune	Luu Minh Commune	Giao Ai Commune	Le Son Nam Commune
Number of residents in the commune eligible for inclusion	3747	719	908	602	1518
Number consenting to study	3049	575	622	546	1306
Number who had serology performed ^+^	3034	570	616	545	1303
Participation rate (%)	81.4	80.0	68.5	90.7	86.0
Age, years					
5–15	626 (20.5)	120 (20.9)	125 (20.0)	95 (17.4)	286 (21.9)
16–35	830 (27.2)	158 (27.5)	128 (20.6)	142 (26.0)	402 (30.8)
36–55	946 (31.0)	158 (27.5)	227 (36.5)	150 (27.5)	411 (31.5)
56–75	539 (17.7)	116 (20.2)	125 (20.0)	126 (23.1)	172 (13.2)
≥76	108 (35.4)	23 (4.0)	17 (2.7)	33 (6.0)	35 (2.7)
Period of serological testing		4 to 13 September	13 to 23 November	6 to 24 November	21 to 23 November
Period of local outbreak		6 to 23 April	25 to 31 July	2–4 July to 1 August	24 July to 9 August
Time from end of outbreak to testing		19 to 20 weeks	16 to 17 weeks	16 to 17 weeks	15 to 16 weeks
Female gender, n (%)	1624 (53.3)	309 (53.7)	336 (54.0)	298 (54.6)	681 (52.1)
Category of exposure					
Household contacts, n (%)	27 (0.9)	8 (1.4)	4 (0.6)	5 (0.9)	10 (0.8)
Close contacts (%)	53 (1.7)	26 (4.5)	10 (1.6)	12 (2.2)	5 (0.4)
Community members (%)	2969 (97.4)	541 (94.1)	608 (97.7)	529 (96.9)	1291 (98.9)
Occupation (%)					
Student *	812 (26.6)	158 (27.5)	153 (24.6)	126 (23.1)	375 (28.7)
Farmer	547 (17.9)	189 (32.9)	69 (11.1)	175 (32.1)	114 (8.7)
Self-employed	438 (14.4)	71 (12.3)	126 (20.3)	60 (11.0)	181 (13.9)
Factory worker	304 (10.0)	43 (7.5)	48 (7.7)	70 (12.8)	143 (10.9)
Office worker	201 (6.6)	9 (1.6)	72 (11.6)	17 (3.1)	103 (7.9)
Retired	108 (3.5)	14 (2.4)	41 (6.6)	25 (4.6)	28 (2.1)
Unemployed	53 (1.7)	3 (0.5)	10 (1.6)	9 (1.6)	31 (2.4)
Service workers	50 (1.6)	3 (0.5)	7 (1.1)	7 (1.3)	33 (2.5)
Health care worker	15 (0.5)	1 (0.2)	11 (1.8)	1 (0.2)	2 (0.2)
Tourist	2 (0.1)	0 (0.0)	0 (0.0)	1 (0.2)	1 (0.1)
Overseas students, returning home	1 (0.0)	0 (0.0)	1 (0.2)	0 (0.0)	0 (0.0)
People returning home	0 (0.0)	0 (0.0)	0 (0.0)	0 (0.0)	0 (0.0)
Other	518 (17.0)	84 (14.6)	84 (13.5)	55 (10.1)	295 (22.6)
Comorbidities (%)					
Smoker	429 (14.1)	89 (15.5)	91 (14.6)	92 (16.8)	157 (12.0)
Hypertension	212 (7.0)	41 (7.1)	50 (8.0)	43 (7.9)	78 (6.0)
Heart disease	85 (2.8)	17 (3.0)	13 (2.1)	18 (3.3)	37 (2.8)
Diabetes	61 (2.0)	18 (3.1)	8 (1.3)	16 (2.9)	19 (1.5)
Renal disease	43 (1.4)	7 (1.2)	8 (1.3)	14 (2.6)	14 (1.1)
Pulmonary disease	30 (1.0)	4 (0.7)	7 (1.1)	4 (0.7)	15 (1.1)
Immunological disease	7 (0.2)	5 (0.9)	0 (0.0)	1 (0.2)	1 (0.1)
Travel history since 1 January 2020 (%)					
Outside Vietnam	1 (0.0)	0 (0.0)	0 (0.0)	0 (0.0)	1 (0.1)
Central Vietnam *	180 (5.9)	0 (0.0)	46 (7.4)	92 (16.8)	42 (3.2)
- Plane			0 (0.0)	1 (0.2)	0 (0.0)
- Bus			2 (0.3)	2 (0.4)	4 (0.3)
- Train			0 (0.0)	0 (0.0)	0 (0.0)
- Private vehicle			44 (7.1)	92 (16.8)	39 (3.0)
- Other			2 (0.3)	1 (0.2)	1 (0.1)
Other local areas	591 (19.4)	104 (18.1)	151 (24.2)	174 (31.9)	162 (12.4)
- Plane		33 (5.7)	15 (2.4)	4 (0.7)	47 (3.6)
- Bus		26 (6.3)	22 (3.5)	13 (2.4)	45 (3.4)
- Train		1 (0.2)	0 (0.0)	0 (0.0)	7 (0.5)
- Private vehicle		58 (10.1)	107 (17.2)	147 (26.9)	87 (6.7)
- Other		2 (0.3)	5 (0.8)	7 (1.3)	3 (0.2)
Symptoms during the local COVID-19 outbreak (%)					
Total	162 (5.3)	33 (5.7)	43 (6.9)	64 (11.7)	22 (1.7)
Fatigue	36 (1.2)	6 (1.0)	6 (1.0)	18 (3.3)	6 (0.5)
Nasal discharge	37 (1.2)	9 (1.6)	15 (2.4)	10 (1.8)	3 (0.2)
Sore throat	36 (1.2)	7 (1.2)	13 (2.1)	10 (1.8)	6 (0.5)
Headache	36 (1.2)	2 (0.3)	10 (1.6)	22 (4.0)	2 (0.2)
Cough	24 (0.8)	9 (1.6)	8 (1.3)	4 (0.7)	3 (0.2)
Myalgia	22 (0.7)	0 (0.0)	3 (0.5)	16 (2.9)	3 (0.2)
Sputum	17 (0.6)	6 (1.0)	5 (0.8)	5 (0.9)	1 (0.1)
Breathlessness	13 (0.4)	6 (1.0)	2 (0.3)	4 (0.7)	1 (0.1)
Diarrhoea	12 (0.4)	6 (1.0)	1 (0.2)	2 (0.4)	3 (0.2)
Fever	9 (0.3)	1 (0.2)	4 (0.6)	3 (0.5)	1 (0.1)
Conjunctivitis	5 (0.2)	3 (0.5)	0 (0.0)	2 (0.4)	0 (0.0)
Anosmia	3 (0.1)	1 (0.2)	1 (0.2)	1 (0.2)	0 (0.0)
Chills	1 (0.0)	0 (0.0)	0 (0.0)	0 (0.0)	1 (0.1)
Haemoptysis	0 (0.0)	0 (0.0)	0 (0.0)	0 (0.0)	0 (0.0)
Other	13 (0.4)	7 (1.2)	2 (0.3)	1 (0.2)	3 (0.2)
Actions taken by those who were symptomatic					
Take pharmacy medications	58 (35.8)	10 (30.2)	20 (46.5)	22 (34.4)	6 (27.3)
Wait	44 (27.2)	7 (21.2)	10 (23.3)	22 (34.4)	5 (22.7)
Take medications at home	25 (15.4)	2 (6.1)	11 (25.6)	11 (17.2)	1 (4.5)
Visit clinic	23 (14.2)	10 (30.3)	4 (9.3)	6 (9.4)	3 (13.6)
Self-isolate	15 (9.3)	1 (3.0)	4 (9.3)	4 (6.3)	6 (27.3)
Already in hospital	3 (1.9)	1 (3.0)	1 (2.3)	1 (1.6)	0 (0.0)
Visit doctor	3 (1.9)	2 (6.1)	0 (0.0)	1 (1.6)	0 (0.0)
Get COVID test	2 (1.2)	0 (0.0)	1 (2.3)	0 (0.0)	1 (4.5)
Other	23 (14.2)	7 (21.2)	5 (11.6)	6 (9.4)	5 (22.7)
No action	0 (0.0)	0 (0.0)	0 (0.0)	0 (0.0)	0 (0.0)
Reported frequency of actions taken by community (%)					
Hand washing					
- Very often	2379 (78.0)	507 (88.2)	470 (75.6)	388 (71.1)	1014 (77.6)
- Sometimes	576 (18.9)	62 (10.8)	108 (17.4)	116 (21.2)	290 (22.2)
- Not at all	100 (3.3)	6 (1.0)	45 (7.2)	45 (8.2)	4 (0.3)
Wearing a mask					
- Very often	2627 (86.2)	523 (91.0)	523 (84.1)	442 (81.0)	1139 (87.2)
- Sometimes	373 (12.2)	47 (8.2)	78 (12.5)	85 (15.6)	163 (12.5)
- Not at all	58 (1.7)	5 (0.9)	22 (3.5)	25 (4.6)	6 (0.5)
Attending social gatherings					
- Very often	526 (17.3)	74 (12.9)	107 (17.2)	302 (55.3)	43 (3.3)
- Sometimes	650 (21.3)	136 (23.7)	127 (20.4)	172 (31.5)	215 (16.5)
- Not at all	1886 (61.9)	365 (63.5)	387 (62.2)	75 (13.7)	1049 (80.3)

^+^ Serology was not collected in 15 individuals who provided consent due to technical difficulties (e.g., difficult access); * Includes school and university students.

**Table 2:** Characteristics of exposure to COVID-19 cases by household and close contacts.

		Cluster			
	Total	Hoi Commune	Luu Minh Commune	Giao Ai Commune	Le Son Nam Commune
Household exposure	27	8	4	5	10
Average number of COVID-19 contacts	2.3	1.0	4.3	2.6	2.5
Average number of encounters *	6	1.1	13.5	5.2	7.3
Average number of hours per exposure	5.3	3.8	4.0	5.8	5.5
Close contact	53	26	10	12	5
Average number of contacts	1.3	1.1	1.7	1.5	1.2
Average number of encounters *	1.5	1.3	2.3	1.6	1
Average number of hours per exposure	2.2	3.7	0.9	1.2	1

* A single encounter is considered significant if >2 h in duration or within a 2 m distance for 15 min.

**Table 3:** Characteristics of health care workers participating in the study.

Characteristic	n
Total number of participants	148
Number who had serology performed	148
Number who had PCR performed *	69 (46%)
Age, years	
5–15	0 (0.0)
16–35	92 (62.2)
36–55	50 (33.8)
56–75	6 (4.1)
≥76	0 (0.0)
Timing of serology	24 to 30 November
Timing of exposure	30 Jan to 26 November
Number of weeks between exposure and serology (median, IQR)	9.4 (5.4—14.8)
Female, n (%)	100 (67.6)
Healthcare facility	
Danang Lung Hospital	59 (39.9%)
Hoa Vang District Hospital	89 (60.1%)
**Exposure history**	
Number of days between first exposure and serology (median, IQR)	117 (115—119)
Number of days between last exposure and serology (median, IQR)	44 (36–50)
Number of days of contact (median, IQR)	40 (30–48.5)
Number of hours of contact per day (median, IQR)	6 (2–8)
**Occupation (%)**	
Nurse	81 (54.4)
Doctor	35 (23.5)
Allied Health	14 (9.4)
Healthcare assistant	10 (6.7)
Laboratory technician	9 (6.0)
**Number of individuals with any infective symptoms since 1 January 2020 (%)**	
Headache	12 (8.1)
Fever	9 (6.0)
Sore throat	9 (6.0)
Rhinorrhoea	8 (5.4)
Dry cough	6 (4.0)
Fatigue	6 (4.0)
Chills	3 (2.0)
Sputum	3 (2.0)
Myalgia	2 (1.3)
Breathlessness	1 (0.7)
Haemoptysis	0 (0.0)
Anosmia/dysgeusia	0 (0.0)
Diarrhea	0 (0.0)
Conjunctival symptoms	0 (0.0)
Other	0 (0.0)
**Comorbidities among those with symptoms**	
Hypertension	7 (4.7)
Diabetes	1 (0.7)
Pulmonary disease	3 (2.0)
Renal disease	1 (0.7)
Heart disease	1 (0.7)
Immunological disease	0 (0.0)

IQR interquartile range, PCR nucleic acid testing. * All health care workers had RT-PCR testing as part of standard workplace requirements between August and November 2020. For the purposes of this study, RT-PCR was performed in symptomatic individuals only.

**Table 4:** Prevalence of seropositivity within high-risk populations, grouped by cluster.

Population	Overall	Hoi Commune	Luu Minh Commune	Giao Ai Commune	Le Son Nam Commune
Overall	13/3034 (0.4%; 95% CI 0.3–0.7%)	6/570 (1.05%; 95% CI 0.5–2.3%)	1/616 (0.2%; 95% CI 0.0–0.9%)	0/545 (0%)	6/1303 (0.5%; 95% CI 0.2–1.0%)
Household contacts	5/27 (18.5%; 95% CI 8.2–36.7%)	4/8 (50.0%; 95% CI 21.5–78.5%)	0/4 (0%)	0/5 (0%)	1/10 (10%; 95% CI 1.8–40.4%)
Close contact	1/53 (1.9%; 95% CI 0.3–9.9%)	1/26 (3.9%; 95% CI 0.7–18.9%)	0/10 (0%)	0/12 (0%)	0/5 (0%)
Community	7/2954 (0.2%; 95% CI 0.1–0.5%)	1/536 (0.2%; 95% CI 0.0–1.1%)	1/602 (0.2%, 95% CI 0.0–0.9%)	0/528 (0%)	5/1288 (0.4%; 95% CI 0.1–0.9%)
Relative risk of infection for contacts versus community members	31.7; 95% CI 10.9–92.0	78.9; 95% CI 9.5–656.0	13.4; 95% CI 0.6–316.2		17.2; 95% CI 2.1–138.3

CI Confidence interval.

**Table 5:** Characteristics of individuals positive for SARS-CoV-2.

Characteristic	Number (%)
Age, years (median, IQR)	35 (22–49)
Household contacts	5 (38.5%)
Close contacts	1 (7.7%)
Community members	7 (53.8%)
**Occupation (%)**	
Student	4 (30.8)
Self-employed	3 (23.1)
Factory worker	2 (15.4)
Farmer	2 (15.4)
Retired	1 (7.7)
**Comorbidities (%)**	
Smoker	1 (7.7)
Heart disease	1 (7.7)
Hypertension	1 (7.7)
Diabetes	0 (0.0)
Renal Disease	0 (0.0)
Pulmonary disease	0 (0.0)
Immunological disease	0 (0.0)
**Reported frequency of actions taken (%)**	
Hand washing	
- Very often	11 (84.6)
- Sometimes	2 (15.4)
- Not at all	0 (0.0)
Mask	
- Very often	12 (92.3)
- Sometimes	0 (0.0)
- Not at all	1 (7.7)
Attending social gatherings	
- Very often	1 (7.7)
- Sometimes	1 (7.7)
- Not at all	11 (84.6)

IQR interquartile range.

## Data Availability

Data is available from the corresponding author on request.

## Supplementary Materials

The following are available online at https://www.mdpi.com/article/10.3390/ijerph18126353/s1, Figure S1: Daily new cases of COVID-19 in Hanoi between May and November 2020, Figure S2: Daily new cases of COVID-19 in Da Nang between March and November 2020, Figure S3: Daily new cases of COVID-19 in Quang Nam between September and October 2020, Table S1: Confirmed COVID-19 case numbers by commune in Da Nang, Table S2: Confirmed COVID-19 case numbers by commune in Quang Nam.
