# Supplementary material for: Sero-Prevalence of SARS-CoV-2 Antibodies in High-Risk Populations in Vietnam
Source: Int J Environ Res Public Health. 2021 Jun 11;18(12):6353. doi: 10.3390/ijerph18126353 (PMC8296183; doi:10.3390/ijerph18126353)
Supplement: Supplementary file 1 [file ijerph-18-06353-s001.zip › ijerph-1247140-supplementary.pdf]

Figure S1: Daily new cases of COVID-19 in Hanoi between May and November 2020

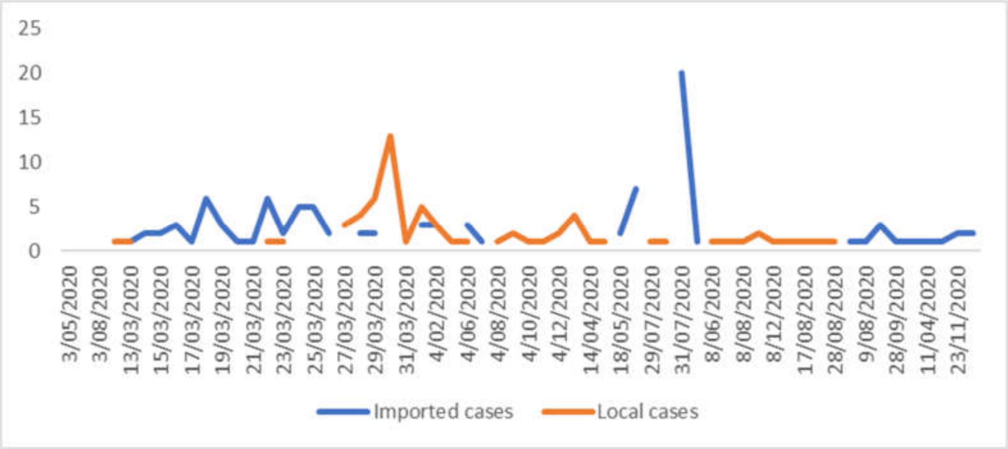

Figure S2: Daily new cases of COVID-19 in Da Nang between March and November 2020

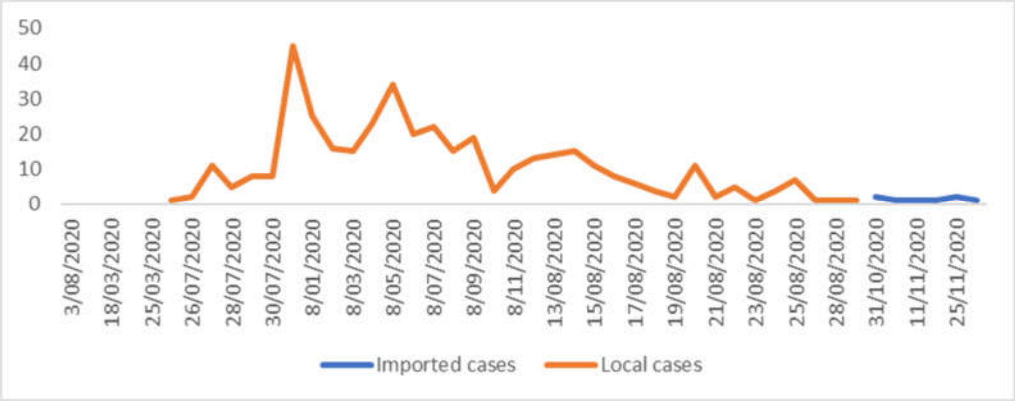

Figure S3: Daily new cases of COVID-19 in Quang Nam between September and October 2020

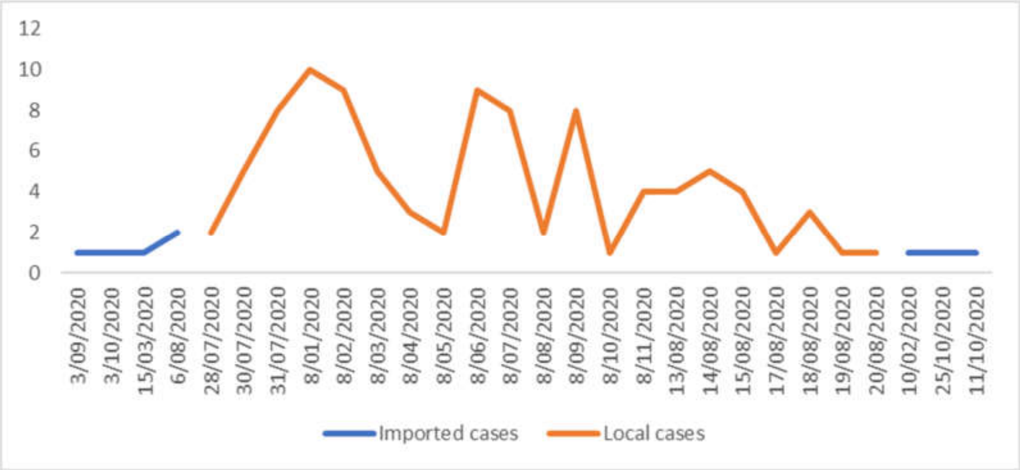

Table S1: Confirmed COVID-19 case numbers by commune in Da Nang

		No. of clusters which had						
**Commune**	**Setting**	**2 cases**	**3 cases**	**4 cases**	**5 cases**	**6 cases**	**7 cases**	**8 cases**
**Hòa Xuân**	Urban	2	1					
**Khuê Trung**	Urban	2						
**Hòa An**	Urban	1						
**Hòa Phát**	Urban		1	1				
**Bình Thuận**	Urban		1					
**Hải Châu 2**	Urban	3						
**Hòa Cường Nam**	Urban		2	1				
**Hòa Thuận Đông**	Urban	1						
**Nam Dương**	Urban		1					
**Phước Ninh**	Urban		1					
**Hòa Thuận Tây**	Urban	1				1		
**Hòa Phong**	Rural	1						
**Hòa Phước**	Rural			1				
**Hòa Tiến***	Rural			1			1	1
**Hòa Khánh Bắc**	Urban	1						
**Hòa Minh**	Urban		1					
**An Hải Bắc**	Urban	2						
**An Hải Đông**	Urban		1		1			

**Nại Hiên Đông**	Urban	3		2				
**Phước Mỹ**	Urban	1	1					
**Thọ Quang**	Urban	1	1					
**An Khê**	Urban	2						
**Chính Gián**	Urban	1						
**Hòa Khê**	Urban	1		1	1			
**Tam Thuận**	Urban		2					
**Tân Chính**	Urban		1					
**Thạch Khê Đông**	Urban	1						
**Thạch Khê Tây**	Urban	1						
**Mỹ An**	Urban					1		

* selected commune

Table S2: Confirmed COVID-19 case numbers by commune in Quang Nam

		No. of clusters which had						
**Commune**	**Setting**	**2 cases**	**3 cases**	**4 cases**	**5 cases**	**6 cases**	**7 cases**	**8 cases**
**Minh An**	Urban						1	
**Cẩm An**	Urban						1	
**Hà Lam***	Urban						1	
**Duy Trung**	Rural			1				
**Điện Hồng***	Rural		1					
**Điện Thọ**	Rural		1					
**Điện Trung**	Rural		1					

* selected commune
